# Numerical Estimation of Limiting Current Density by Focusing on Mass Transfer within Porous Spacers in an Electro-Dialysis

**DOI:** 10.3390/membranes9070075

**Published:** 2019-06-28

**Authors:** Yoshihiko Sano, Kosuke Fukagawa, Fujio Kuwahara

**Affiliations:** Department of Mechanical Engineering, Shizuoka University, 3-5-1 Johoku, Naka-ku, Hamamatsu 432-8561, Japan

**Keywords:** porous spacer, limiting current density, numerical simulation, ion mass transport

## Abstract

Estimating and increasing limiting current density (LCD) levels is of fundamental importance for the development of electrodialysis (ED) systems, and it is becoming clear that the use of porous spacers can significantly increase such LCD levels. In this study, a three-dimensional numerical simulation was proposed for evaluating the mass transfer within a porous spacer unit cell and for estimating LCD levels. It was found that our proposed method is effective for estimating the minimum value of an LCD, which is a significant factor related to the safe operation of ED systems. Furthermore, it was found that increasing the minimum effective Sherwood number provides a key to increasing LCD levels. Porous spacer design guidelines were proposed based on the numerical simulation results, after which a new spacer was introduced, designed according to those guidelines. It was found that flow disturbances on the membrane caused by porous spacer structures can lead to increases in effective Sherwood numbers and that LCD levels could be increased by eliminating the flow stagnation behind the structures on the membrane. The LCD of our new spacer was found to be higher than that of the spacers with the highest LCD levels in use at present. Therefore, we can conclude that the proposed design guidelines are effective for increasing LCD levels.

## 1. Introduction

Electrodialysis (ED) utilizing ion transport through cation and anion exchange membranes, in which electrically charged ions pass through those membranes due to the influence of electrical potential differences, makes it possible to desalinate water and concentrate target minerals in ionic solutions. In review studies [1,2], some advantages of ED, such as a higher removal efficiency for ion species than can be achieved by the reverse osmosis technique, were reported. ED has also been adopted for salt production [3,4], the desalination of brackish water and seawater [5,6], and the removal of heavy metals from industrial wastewater [7,8,9].

Electrophoresis is the main transport phenomenon that occurs when ions pass through an ion exchange membrane, which means that the mass transfer rate per unit of membrane area for electrically charged ions depends on the current density, which is based on Faraday’s law. Therefore, when used in industrial systems, operations with high current density levels are required to produce high concentration levels and dilution rates. However, an upper limiting value exists for current densities in actual ED operations, which is called the “limiting current density (LCD)”, and estimating the LCD is of fundamental importance for operating under optimal conditions in an ED system. Therefore, it is necessary to understand the ion transport phenomena that occur during ED, which are responsible for the LCD of the ED system. In general, ion concentrations decrease in membrane boundary layers in relation to the current density levels. This is called concentration polarization. When ions within a boundary layer on a membrane are depleting, the electrical resistance increases drastically, and finally water dissociation occurs. Furthermore, it is known that electrical interfacial phenomena, including electroconvection, occur. For details, refer to the literature [10]. These issues lead to an increasing energy consumption and the damage of expensive membrane. Thus, a method that could be used to increase LCD levels would make operations with higher concentration levels and dilution rates possible.

Several studies have investigated LCD increases from various viewpoints, such as by applying anon-stationary electric fields [11], air sparging [12], and the use of asymmetric bipolar membranes [13]. Recently, Sano et al. [14] introduced porous spacers for increasing LCD levels and reported that, as a result of the mechanical dispersion caused by fluid mixing in porous materials, the use of their spacers resulted in LCD levels 1.8 to 3.3 times higher than those that were achievable without porous spacers.

Furthermore, Bai et al. [15] investigated the effect of porous spacer structures on the performance for ED desalination by using a three-dimensional (3D) printer to fabricate such spacers. According to their studies [14,15], the role of a porous spacer is to use flow mixing to supply ions directly to the boundary layer on a membrane so that the formation of concentration polarization can be suppressed, in comparison to a conventional net spacer [16,17,18]. In another approach, Balster et al. proposed the use of a multi-layer net spacer [19] and a membrane with an integrated spacer [20]. Taken together, these studies show that the spacer structure is a matter of importance when working to increase LCD levels, which indicates that a simple method for evaluating the effectiveness of a spacer structure on the LCD is required.

With these points in mind, this manuscript focuses on the results of numerical simulations conducted to evaluate ion mass transport within porous spacers and to estimate their LCD levels. While some researchers have previously conducted numerical simulations aimed at evaluating the mass transfer in an ED with net spacers [21,22,23], those studies adopted large computational domains that included entire or large parts of the net spacer, which meant that the calculations became complicated and calculation costs were enormous due to the numerous meshes. Furthermore, the small number of grids around spacer structures makes it difficult to estimate the boundary layers, which leads to the possibility that the calculation precision will deteriorate. Therefore, it is desirable to accurately calculate LCDs while minimizing the calculation costs. In contrast, Nikonenko et al. [10] reviewed the current state of the art associated with the over LCD; moreover, Uzdenova [24] conducted a numerical simulation for evaluating the overlimiting transfer enhanced by electroconvection. According to Uzdenova [24], the electroconvection has to be taken into account to evaluate ion transport phenomena in cases of over LCD, while the effect of electroconvection can be negligible when running under a current density lower than the LCD. Although the electroconvection is important for evaluating ion transport phenomena in the over LCD, the calculation becomes complicated. Therefore, it is desirable to estimate LCDs through the simple method.

In this research, 3D numerical simulations were carried out to evaluate the mass transfer within a porous spacer unit cell, and a method for estimating LCD levels from ion concentrations within a porous spacer was proposed. In our numerical simulations, three porous spacer types proposed by Bai et al. [15], which have the highest LCD levels at present, were evaluated in efforts to confirm our proposed method. Furthermore, the ion concentrations and Sherwood numbers on the ion exchange membranes were evaluated in order to discuss the relationship between the LCD levels and ion transport within the porous spacers. We then propose a new spacer that can provide improved LCD levels based on a set of numerical simulation results.

## 2. Numerical Simulations

### 2.1. Calculation Domain and Porous Spacers

**Figure 1:**
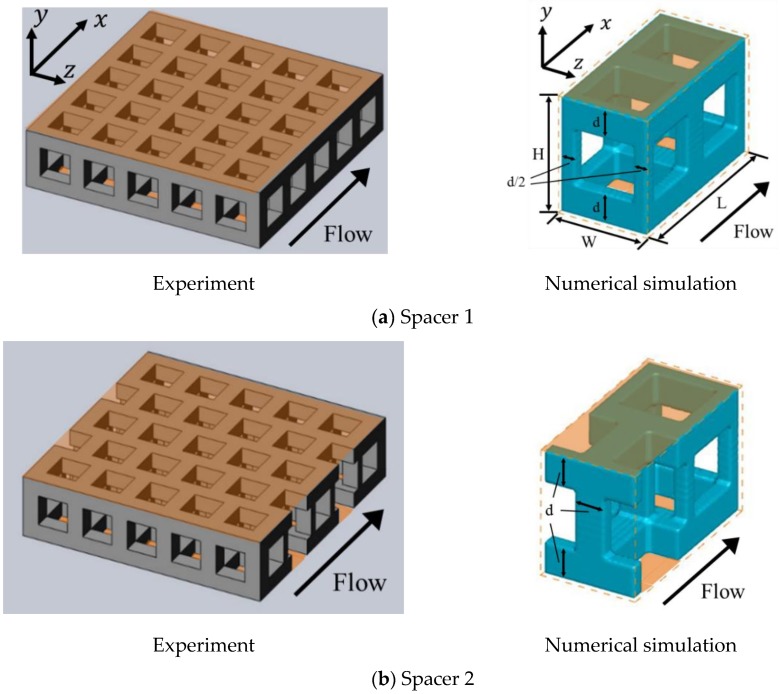

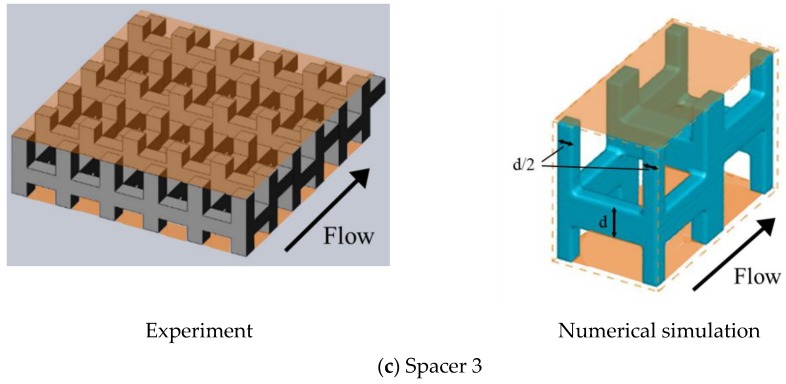
Porous spacer shapes. (**a**) Spacer 1 has a basic structure in which cubic lattices are simply combined with each other; (**b**) Spacers 2 has a staggered cubic lattice arrangement, which is shifted by a half lattice in the *z* direction relative to Spacer 1; (**c**) Spacers 3 has a staggered cubic lattice arrangement, which is shifted by a 1/3 lattice in the *y* direction relative to Spacer 1.

Bai et al. [15] utilized porous spacers composed of a cubic lattice fabricated by a 3D printer to enhance flow mixing. They stated that flow towards the ion exchange membrane when interposing spacers could suppress the development of concentration polarization and thus increase the LCD levels. Figure 1 shows the structures of the porous spacers used in their study, as well as the computational domains applied in this study. The ion exchange membranes, which are shown in orange, are arranged at the upper and lower ends of the spacers, while the characteristics of the porous spacers are listed in Table 1. In our numeral simulations, the calculation domain was the region with a porous spacer unit cell. Spacer 1 (see Figure 1a) has a basic structure in which cubic lattices are simply combined with each other, while both Spacers 2 (see Figure 1b) and 3 (see Figure 1c) have staggered cubic lattice arrangements. Spacer 2 is shifted by a half lattice in the *z* direction relative to Spacer 1, while Spacer 3 is instead shifted by a 1/3 lattice in the *y* direction relative to Spacer 1.

In these numerical simulations, we focused on mass transfer in the dilute phase, since this is the phase where dilute concentration polarization occurs and thus the phase that impacts LCD levels. This focus was further limited to a central unit in the channel width in order to prevent the influence of the gasket wall from impacting the calculations. By adopting the calculation domain explained above, it is possible to adopt a fine computational grid for each unit structure, as compared to those described in other studies, which means that the flow and concentration fields can be accurately calculated, even against more complicated structures. Additionally, the number of computational grids was set to 60 in the *x* direction (i.e., the main flow direction) and *y* direction (i.e., the membrane direction), while it was set to 30 in the *z* direction. Furthermore, unequally spaced computational grids were applied in order to calculate the concentration boundary layer on the membrane, and the width of the finest computational grid was set to 0.05 mm.

### 2.2. Governing Equations and Mass Balance in Solution

Usually, the Reynolds number of a flow within the ED channels is small, so the movement can be considered as a laminar flow. Continuity and Navier-Stokes equations for the steady and incompressible fluids are given as follows:

(1) $$\frac{\partial u_{j}}{\partial x_{j}}=0$$

(2) $$\rho \frac{\partial u_{j}u_{i}}{\partial x_{j}}=-\frac{\partial p}{\partial x_{i}}+\mu \frac{\partial }{\partial x_{j}}\left(\frac{\partial u_{i}}{\partial x_{j}}+\frac{\partial u_{j}}{\partial x_{i}}\right)$$

where *u_i_*, *ρ*, *p*, and *μ* are the velocity vector, density, pressure, and viscosity, respectively. Nakayama et al. [25] derived a diffusion equation from the Nernst-Planck equation under a local electro-neutrality assumption, as follows:

(3) $$\frac{\partial u_{j}c}{\partial x_{j}}=\frac{2D_{1}D_{2}}{D_{1}+D_{2}}\frac{\partial^{2}c}{\partial x_{j}{}^{2}}$$

The simple diffusion formula, shown in Equation (3), can handle the ion mass transport, thereby canceling the electrophoresis term in Nernst’s equation. Therefore, this diffusion term is considered as the diffusion, including the ion molar flux of the original diffusion and electrophoresis. However, there are restrictions on the establishment conditions. This formula is established in an ionic solution with one pair of cation and anion ions with the same valence electron number. In this study, we deal with a NaCl solution that satisfies this condition. Incidentally, a NaCl solution was also used in the experiment that will be used for comparison purposes in this study. Therefore, the relationship of the molar concentrations *c* = *c*_1_ = *c*_2_ is given based on local electro-neutrality, where subscripts 1 and 2 indicate Na^+^ and Cl^−^, respectively. Two diffusion coefficients, *D*_1_ = 1.33 × 10^−9^ m^2^/s and *D*_2_ = 2.03 × 10^−9^ m^2^/s, were applied in the numerical simulation. Bai and Nakayama [23] adopted this equation in their numerical simulation, which used a large computational domain (including large parts of the net spacer), but this calculation is still under development in terms of large calculation domains and boundary conditions.

In contrast, the computational domain used in this study was a porous spacer unit cell. Additionally, a periodic boundary condition was adopted at the boundary (*x* = 0, *L*) in the *x* direction to facilitate accurate predictions of flow and concentration within the porous spacer, as follows:

(4) $$u{|}_{in}=u{|}_{out}$$

(5) $$c{|}_{in}=c{|}_{out}+\Delta c_{B}$$

where *Δc_B_* is the concentration difference between the inlet and outlet of a porous spacer unit cell, which can be obtained from the following mass balance equation by assuming the constant current density condition:

(6) $$A_{x}u_{B}\Delta c_{B}=2A_{y}q$$

where *u_B_* is the intrinsic mean flow velocity. On the other hand, *A_x_* represents the cross-sectional intrinsic area (i.e., the area of spacer that is excluded) at a boundary in the main flow direction, while *A_y_* is the intrinsic area of the membrane, respectively. In other words, *A_y_* means the area of the total area of the membrane *A_mem_* (= *W* × *L*) minus the area in contact with the membrane and spacer, which is also shown in Table 1. Moreover, *q* is the ion flux passing through the membrane, which is calculated based on Faraday’s law, as follows:

(7) $$q=\frac{i}{2F}\frac{A_{mem}}{A_{y}}$$

where *F* and *i* are Faraday’s constant and the current density, respectively. In this study, in order to deal with the ideal ion transport, it was assumed that each transport number in the ion exchange membranes is 1; moreover, each transport number in solution is 0. This current density *i* is the value usually used in our experiment. Specifically, it is the current divided by the entire membrane area. Usually, ions pass from the solution through the membrane, but they cannot pass at locations where the membrane and spacer are in contact. Thus, the local current density at locations where the membrane and spacer are not in contact should be higher than the current density *i*. In Equation (7), the ion flux passing through the membrane is estimated by multiplying the ratio of the total membrane area with the intrinsic membrane area. Therefore, in this numerical simulation, the ion transport boundary condition on the membrane was given as follows:

(8) $$-\frac{2D_{1}D_{2}}{D_{1}+D_{2}}\frac{\partial c}{\partial y}=q=\frac{i}{2F}\frac{A_{mem}}{A_{y}}$$

Furthermore, the velocity was converged to the target mean flow velocity by imposing a restraint condition against the flow rate at the inlet and outlet boundaries. Symmetrical boundary conditions were imposed on the boundaries in the *z* direction, while non-slip conditions were given at the boundaries in the *y* direction (i.e., on membranes, *y* = ± *H*/2). On the other hand, the bulk concentration at the inlet boundary was converged to the constant value by imposing a restraint condition so as to avoid numerical divergence. In other words, a reference point for calculating the ion concentration was given at the inlet boundary.

Based on the assumption of the constant current density condition, the concentration distribution in a porous spacer unit cell is similar to that in the spacer at any position, in this case at the given current density. In this numerical simulation, the LCD level is measured based on this similarity. Of course, this similarity may not be applied to the entrance of the ED stack where the flow is not developed and to the condition where the electric convection occurs. In this study, we assumed that these situations can be ignored in order to propose a simple method to determine the LCD level.

Furthermore, in this study, the above governing equations and boundary conditions were normalized to obtain more versatility solutions. This normalization makes us solve the problem regarding the fact that the current density has to be imposed in the boundary condition despite determining the LCD level. To normalize the above equations, the following dimensionless variables were defined:

(9) $$u_{ref}=u_{B},\;L_{ref}=H,\;\Delta C_{ref}=\frac{2q}{u_{B}}\frac{A_{y}}{A_{x}}$$

Exploiting these dimensionless variables, the governing equations were normalized as follows:

(10) $$\frac{\partial u^{*}{}_{j}}{\partial x^{*}{}_{j}}=0$$

(11) ∂u*ju*i∂x*j=−∂p*∂x*i+1Re∂∂xj(∂u*i∂x*j+∂u*j∂x*i)

(12) ∂u*jc*∂x*j=1ReSc∂2c*∂x*j2

where *Sc* is the Schmidt number, defined as follows:

(13) $$Sc=\frac{\nu }{\frac{2D_{1}D_{2}}{D_{1}+D_{2}}}$$

Furthermore, the boundary conditions were normalized as follows:

(14) $$u^{*}{|}_{in}=u^{*}{|}_{out}$$

(15) $$c^{*}{|}_{in}=c^{*}{|}_{out}+1$$

(16) −∂c*∂y*=12AxAyReSc

Therefore, in this numerical simulation, the dimensionless concentration distribution in a porous spacer unit cell can be obtained without a current density, simply giving the dimensionless geometric dimension of the porous spacer, Reynolds number (*Re* = 28~140, where *ν* = 0.893 × 10^−6^ m^2^/s) and Schmidt number (*Sc* = 556) based on the experimental conditions. 

### 2.3. Calculating the Sherwood Number and Limiting Current Density

We shall consider the estimation of the Sherwood number. The ion mass flux can also be given by Newton cooling’s law, as follows:

(17) $$q=\frac{A_{mem}}{A_{y}}h_{eff}\left(c_{W}-c_{B}\right)$$

where *h_eff_* is defined as the effective mass transfer coefficient, which is the mass transfer coefficient when the same ion mass flux is imposed on the local membrane. By introducing the effective mass transfer coefficient, we can discuss the effect of flow within porous spacers on the ion mass transport regardless of the current density level. Furthermore, subscripts *W* and *B* indicate the membranes’ surface and the bulk value of the vertical cross-section against the main flow, respectively. Substituting Equation (17) into Equation (8), we obtain:

(18) $$-\frac{2D_{1}D_{2}}{D_{1}+D_{2}}\frac{\partial c}{\partial y}=\frac{A_{mem}}{A_{y}}h_{eff}\left(c_{W}-c_{B}\right)$$

Rewriting the above equation with an effective Sherwood number that can evaluate the ion mass transport on the membrane, we obtain:

(19) $$Sh_{eff}=\frac{h_{eff}H}{\frac{2D_{1}D_{2}}{D_{1}+D_{2}}}=-\frac{A_{y}}{A_{mem}}\frac{H}{\left(c_{W}-c_{B}\right)}\frac{\partial c}{\partial y}=-\frac{A_{y}}{A_{mem}}\frac{1}{\left(c^{*}{}_{W}-c^{*}{}_{B}\right)}\frac{\partial c^{*}}{\partial y^{*}}$$

**Figure 2:**
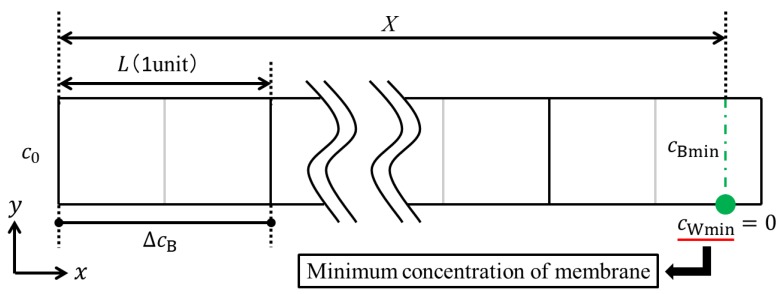
Limiting current density occurrence point. A point where water dissociation is occurring is located at the end of a channel in the dilute phase. *c_Bmin_* and *c_Wmin_* are indicated as the minimum bulk concentration and the minimum concentration on the membrane, respectively. Furthermore, *c*_0_ and *X* are the initial concentration and length of the dilute channel, which are given from experimental condition.

Next, we shall consider the determination of the LCD. Assuming that water dissociation occurs at the location where the minimum concentration on the membrane becomes zero, the current density at which water dissociation occurs is defined as the LCD. As shown in Figure 2, a point where water dissociation occurs would be located at the end of a channel in the dilute phase, since the concentration in the point is lowest in an ED cell. In this illustration, *c_Bmin_* and *c_Wmin_* are indicated as the minimum bulk concentration and the minimum concentration on the membrane, respectively. The minimum bulk concentration refers to the bulk concentration when water dissociation occurs. Therefore, the mass balance in the whole channel can be given as follows:

(20) $$c_{0}-\frac{X}{L}\Delta c_{B}=c_{B\min}=c^{*}{}_{B\min}\Delta c_{B}$$

Substituting Equations (7) and (20) into Equation (6), we obtain:

(21) $$LCD=Fu_{B}\frac{A_{x}}{A_{mem}}\frac{c_{0}}{\frac{X}{L}+c^{*}{}_{B\min}}$$

where c*_Bmin_ is the dimensionless minimum bulk concentration when water electrolysis occurs. Thus, it is necessary to correct the concentration distribution in a porous spacer unit cell so that the minimum ion concentration on the membrane becomes zero (i.e., *c_Wmin_* = c*_Wmin_ = 0). Based on the similarity of the calculated concentration distribution in a porous spacer unit cell, the correction value c*_cor_, which is calculated by c*_cor_ + c*_Wmin_ = 0, can be added to the dimensionless concentration distribution. After this correction, the LCD can be determined from Equation (21) by substituting c*_Bmin_ and the other values given from the experimental condition or equipment. In this numerical simulation, *c*_0_ and *X* are the initial concentration and length of the dilute channel, which were given as 342 mol/m^3^ (2.0% W/W) and 0.6 m in the previous experiment. The procedures to determine the LCD are summarized as follows:

1. Finding the dimensionless minimum concentration on the membrane c*_Wmin_ after the numerical integration with Equations (10)–(12).
2. Determining the correction value c*_cor_ so that the dimensionless minimum concentration on the membrane becomes zero.
3. Adding the correction value c*_cor_ to the entire calculation region, and estimating the dimensionless minimum bulk concentration c*_Bmin_.
4. Determining the value of the LCD with Equation (21).

In this numerical simulation, direct numerical integrations of Equations (10) to (12) were carried out via the finite volume method, and a simple method was adopted for the pressure correction. Furthermore, the dimensioned concentration was estimated from Equation (9) and the determined LCD. In this manuscript, for the reader’s understanding, we show the concentration distribution with the dimension of %W/W estimated by this process.

## 3. Results and Discussion

**Figure 3:**
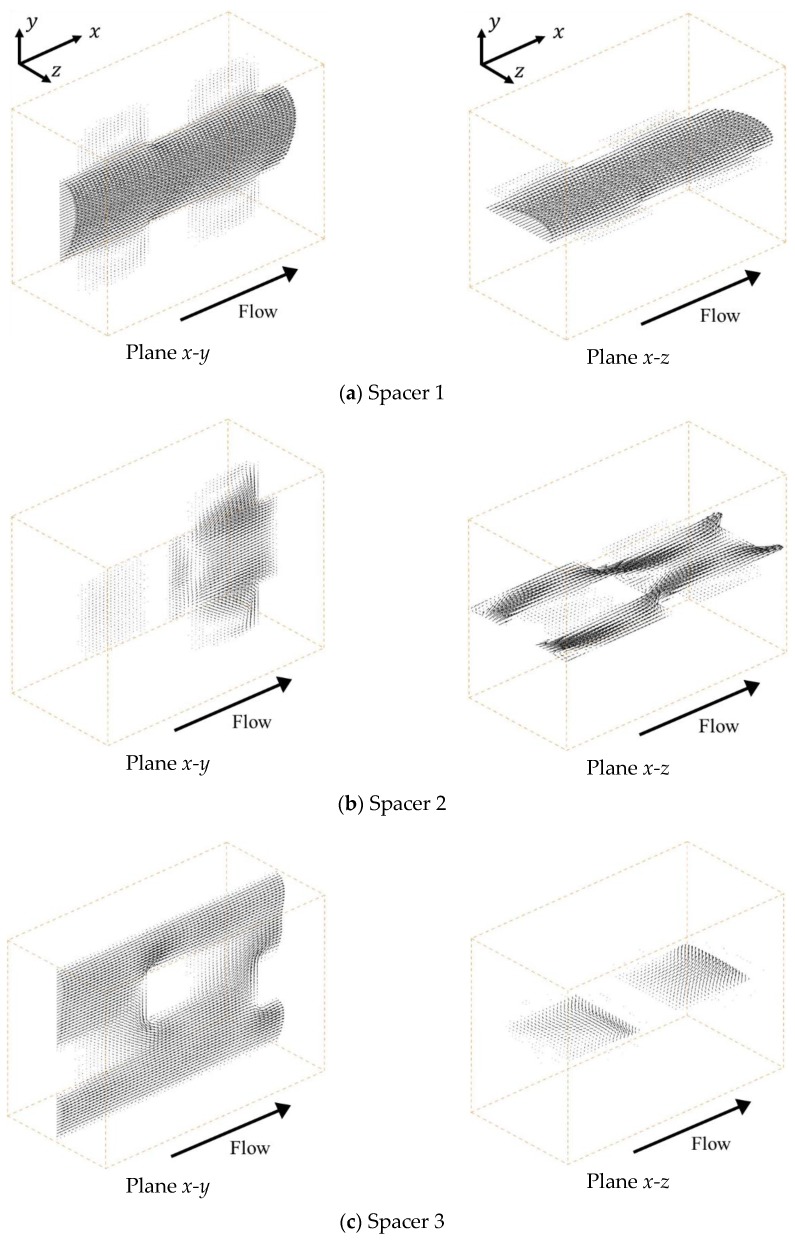
Velocity fields with porous spacers. (**a**) The flow is mainly linearly at the center part of the spacer and is shaped similar to a parabolic stream; (**b**) The flow is repeatedly separated and mixed in parallel to the ion exchange membranes due to the existence of Spacer 2; (**c**) The flow is repeatedly separated and mixed in the direction relative to the ion exchange membrane due to the existence of Spacer 3.

Figure 3 shows the velocity vectors under a mean flow velocity of 1.5 cm/s at the centers of the *x-y* and *x-z* planes, respectively. In the case of Spacer 1, as shown in Figure 3a, the ionic solution flow is mainly linearly at the center part of the spacer and is shaped similar to a parabolic stream, such as a Hagen-Poiseuille flow. This is because Spacer 1 is a square arrangement structure produced simply by combining cubic lattices and is arranged so that the flow path is linear at the center part. Furthermore, it can be seen that there is a secondary flow toward the ion exchange membrane that is weaker than the main stream. 

On the other hand, in the case of Spacer 2, as shown in Figure 3b, the main flow is repeatedly separated and mixed in parallel to the ion exchange membranes due to the existence of the porous spacer. As previously mentioned, Spacer 2 has a staggered arrangement that is shifted by a half lattice in the *z* direction relative to Spacer 1, which causes the flow to meander parallel to the membrane. Furthermore, low-velocity fields are formed between the neighboring structures in the *x* direction. In the case of Spacer 3, as shown in Figure 3c, the flow is also repeatedly separated and mixed in the direction relative to the ion exchange membrane. Additionally, as explained above, Spacer 3 has a staggered arrangement that is shifted alternately by a 1/3 lattice in the *y* direction relative to Spacer 1. By shifting the structure by a third in the *y* direction, the ion solution is stirred more thoroughly with Spacer 3 than with Spacer 2 because the same structure does not appear continuously in the *y* direction. However, although the flow velocity on the membrane is higher than that of other spacers, a developed flow seems to exist on the membrane. 

**Figure 4:**
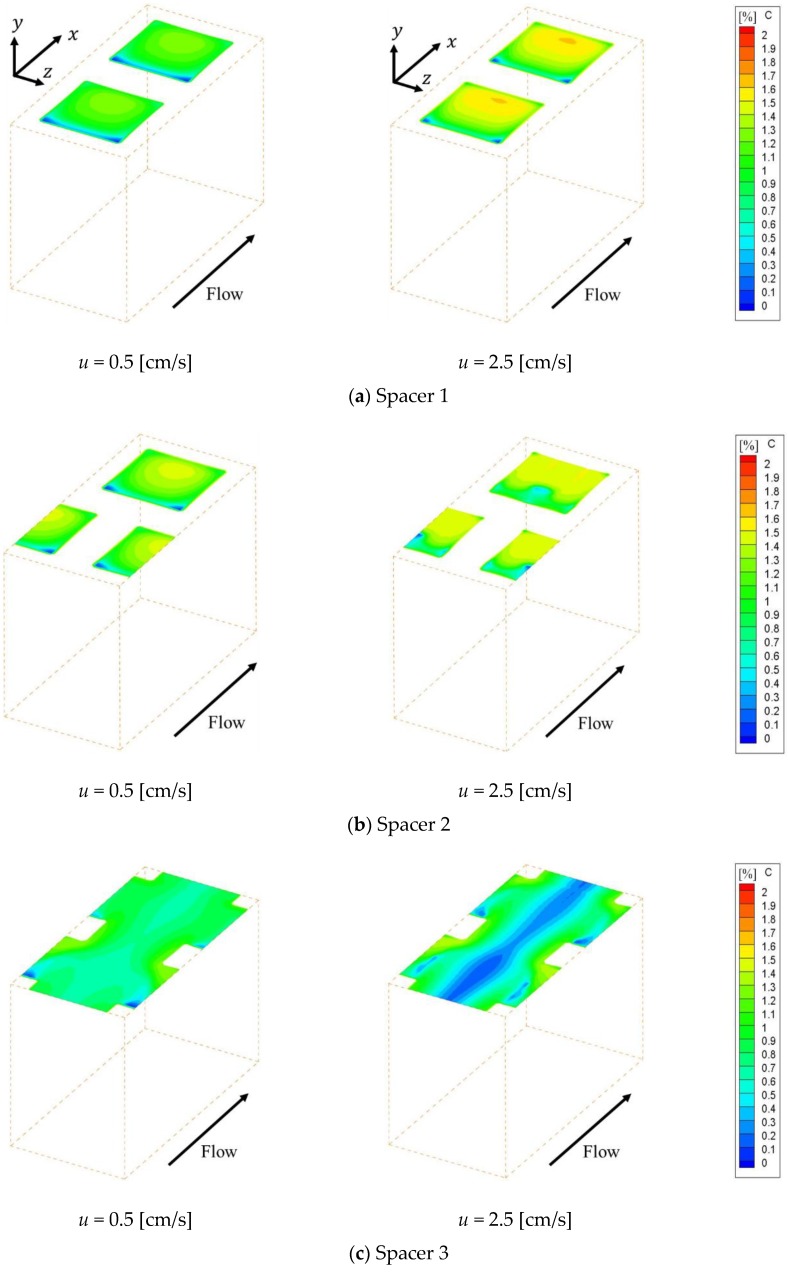
Concentration fields on an ion exchange membrane with porous spacers. (**a**,**b**) High concentration regions are formed at the attachment point of recirculation flow, while the low concentration regions are formed behind the structure; (**c**) Low concentration regions are observed on the center of the membrane along the main flow, which was not seen from (**a**,**b**).

Figure 4 shows the concentration fields on an ion exchange membrane under the velocities of 0.5 cm/s and 2.5 cm/s, which are the minimum and maximum values of the previous experiment, respectively. Figure 4a–c shows the concentration fields within Spacer 1, 2 and 3 respectively. Note that the data in Figure 4 have already been corrected so that the minimum concentration on the membrane becomes zero. As can be seen from the figure, the ion concentration on the membrane decreases behind the structure, which is a location where the flow is stagnant. The concentration is subject to a decrease in places where the supply of ions supplied by convection is depleted. In contrast, in the case of Spacer 3, ion concentration decreases were observed on the center of the membrane along the main flow, which was not seen in the cases of Spacers 1 and 2. The solution at the membrane center flows straight in the *x* direction, while the flow near the structure is distorted due to the presence of obstacles as it approaches the structure. The boundary layer becomes thinner where the flow direction changes, and diffusion increases the ion concentration on the membrane in comparison to the level seen on the central part.

**Figure 5:**
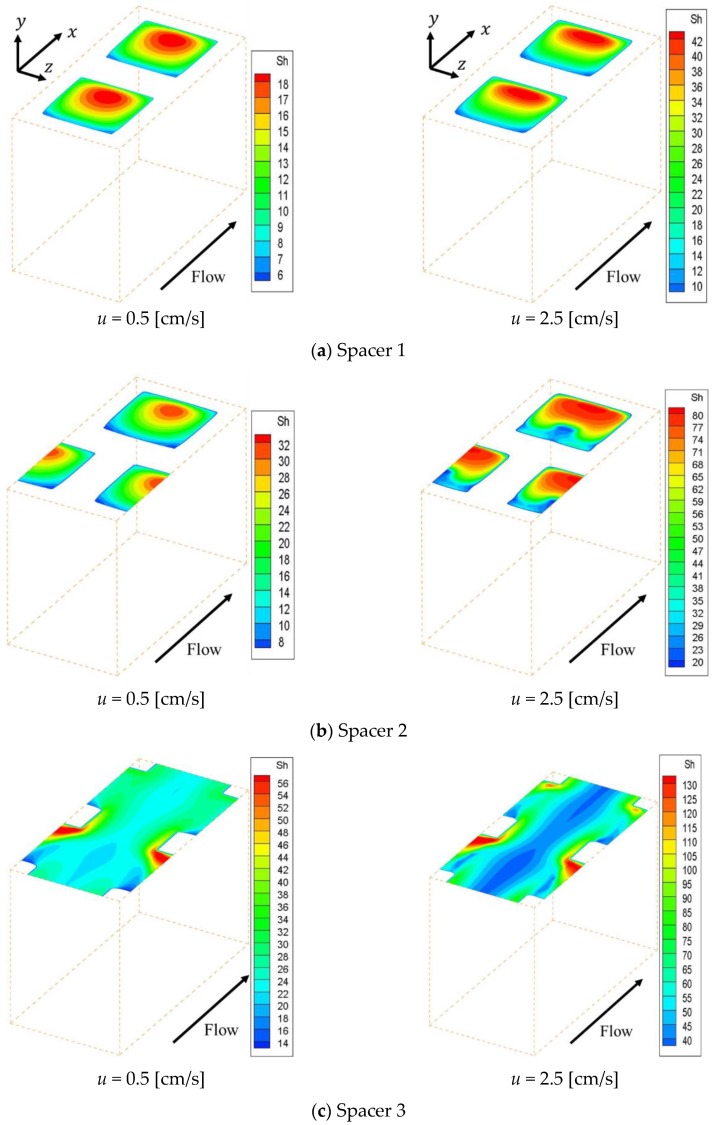
Effective Sherwood number distribution on the ion exchange membrane. The effective Sherwood number distribution is correlated with the concentration distribution on the membrane. (**a**,**b**) The effective Sherwood numbers are highest at the circulating flow reattachment points; (**c**) The effective Sherwood number is highest where the upward flow generated by the structure inside the channel collides with the structure on the membrane. Furthermore, the effective Sherwood number is not high at the center of the membrane, where the boundary layer thickness is not thin because a straight flow exists.

Figure 5 shows the effective Sherwood number distribution on the ion exchange membrane under the velocities of 0.5 cm/s and 2.5 cm/s. Figure 5a–c shows the effective Sherwood number distributions within Spacer 1, 2 and 3 respectively. This figure indicates that the effective Sherwood number under the 2.5 cm/s condition is higher than that of the 0.5 cm/s condition for the whole membrane. Furthermore, it can be seen that the effective Sherwood number distribution is correlated with the concentration distribution on the membrane shown in Figure 4. In other words, the effective Sherwood number is high at places where the ion concentration is high and low at places where the ion concentration is low. 

In general, the boundary layer thins at locations where the effective Sherwood number is high. Therefore, the mass transfer of ions toward the membrane is high at places with high Sherwood numbers, which increases the concentration on the membrane itself. As can be seen from Figure 5, the effective Sherwood numbers are low behind the structures in all spacers. We can also see that, in the cases of Spacers 1 and 2, the effective Sherwood numbers are highest at the circulating flow reattachment points that occur between the structures on the membrane. In contrast, in the case of Spacer 3, the effective Sherwood number is highest where the upward flow generated by the structure inside the channel collides with the structure on the membrane. Furthermore, the effective Sherwood number is not high at the center of the membrane, where the boundary layer thickness is not thin because a straight flow exists.

**Figure 6:**
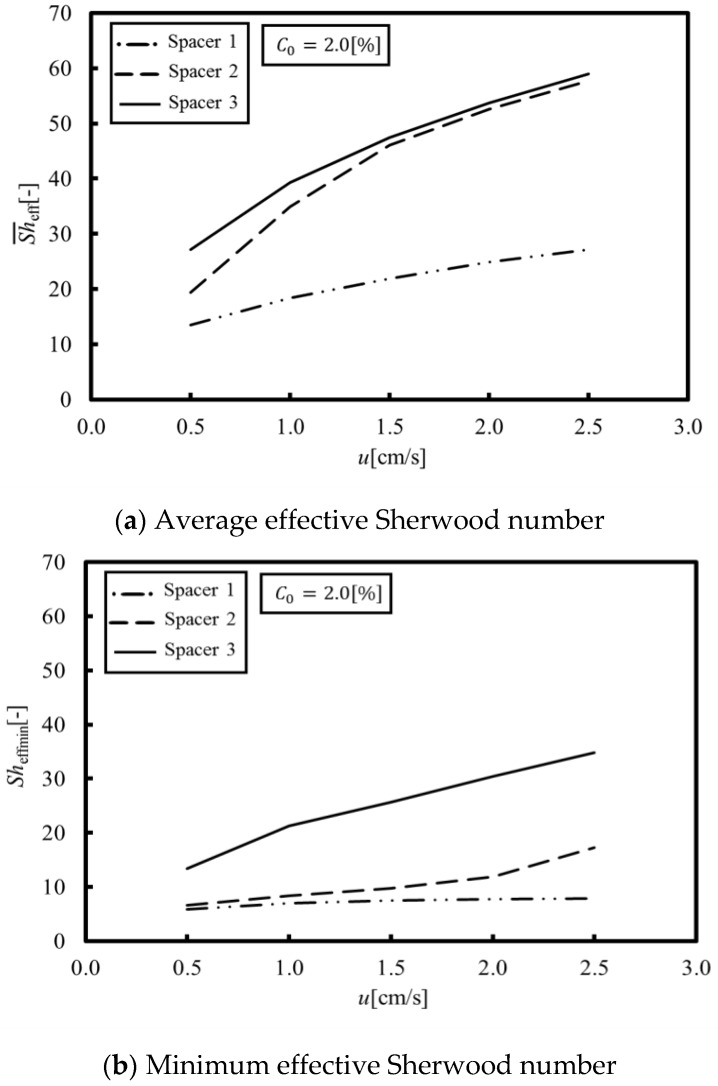
Average and minimum effective Sherwood numbers. (**a**) The average effective Sherwood numbers of Spacers 2 and 3 are much higher than that of Spacer 1; (**b**) The minimum effective Sherwood number of Spacer 3 is the highest in all of the tested spacers.

Figure 6a,b shows the average and minimum effective Sherwood numbers obtained from a set of numerical simulations under velocities within 0.5 to 2.5 cm/s, where the average effective Sherwood numbers means the averaged value of the local Sherwood numbers on the membrane over a porous spacer unit cell. As can be seen in Figure 6a, the average effective Sherwood numbers of Spacers 2 and 3 are much higher than that of Spacer 1, which indicates that the average effective Sherwood number can be increased, even in a circulating flow on a membrane, by agitating the main stream with a porous spacer. On the other hand, as shown in Figure 6b, the minimum effective Sherwood number of Spacer 3 is the highest in all of the tested spacers. This can be explained by flow stagnation. More specifically, in the case of Spacers 1 and 2, the flow stagnation occurs behind the membrane structures because the recirculation flow occurs on the membrane itself. In contrast, in the case of Spacer 3, the flow stagnation is less than for Spacer 1 and 2 because the main stream flows along the membrane.

**Figure 7:**
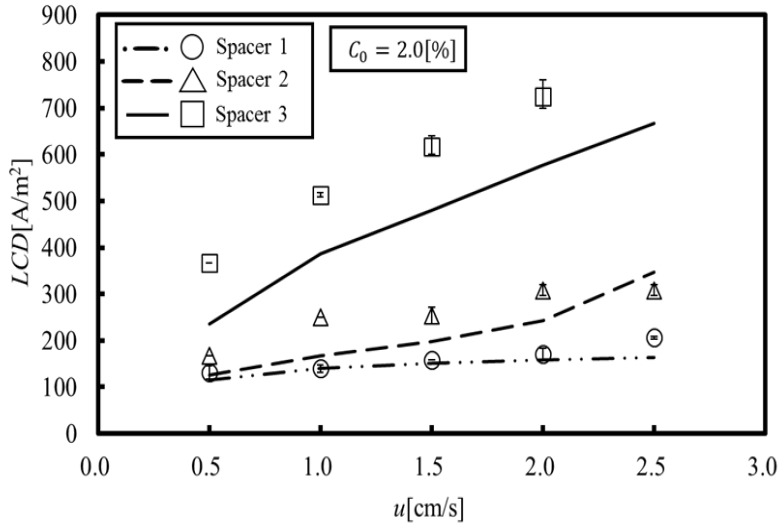
Limiting current density (numerical simulation and experiment [15]). The LCD obtained by our simulation reproduces the experimental results for Spacers 1 and 2. A comparison between LCD and the minimum effective Sherwood number shows correlations between them.

Figure 7, in which the experimental data are also plotted for comparison, shows the LCD obtained by the proposed method. The experimental data were measured by the pH value changes when water dissociation occurred [15]. It can be seen that the LCD obtained by our simulation reproduces the experimental results for Spacers 1 and 2. In Spacers 1 and 2, the location showing the minimum concentration is formed in a relatively wide area behind the structure on the membrane. Thus, it is thought that the LCD was easily estimated because water dissociation occurs within this wide area. On the other hand, it can be seen that the LCD obtained by our simulation qualitatively reproduces the experimental results for Spacers 3. The minimum concentration in Spacer 3 occurs at a small point behind the structure on the membrane, and even if water dissociation occurs at this point, it would probably be difficult to measure the LCD from the resulting pH values. In other words, the proposed method predicts a minute water dissociation and estimates the minimum value of an LCD, which is a significant factor related to the safe operation of ED systems.

On the other hand, a comparison between Figure 6b and Figure 7 shows correlations between them, since the LCD is estimated at the points where the concentration is the lowest. This indicates that suppressing the local concentration reduction, specifically by increasing the minimum effective Sherwood number, provides a key to increasing the LCD. Based on the above discussions, the following three points are introduced as factors relevant for increasing the LCD:

(a) Using the structure to agitate the main stream flows on the membrane in order to increase the effective Sherwood number;
(b) Using the structure to form the flow toward the membrane, thereby mixing the solution within the interior and outside of the boundary layer in order to facilitate ion consumption from the whole channel; and
(c) Reducing the contact area between the structure and the membrane in order to eliminate flow stagnation behind the structure; which should be done to ensure that the membrane is not damaged by the structure.

However, while we can and should consider ways to eliminate the structure on the membrane, the spacer needs to be in contact with the membrane in order to fulfill its designed role of creating space between the membranes. If we were to eliminate membrane structures completely, the boundary layer would be thickened by the straight flow occurring along the membrane.

**Figure 8:**
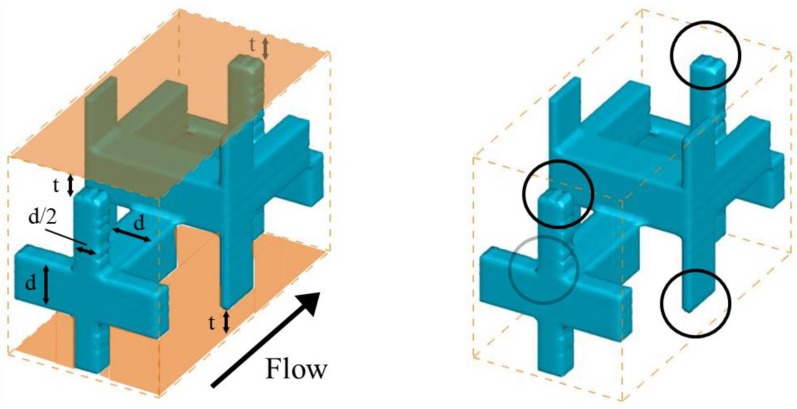
Spacer 4 structure, which has the geometric characteristics of *ε* = 0.81, *A_y_/A_mem_* = 0.97 and *A_x_* = 11.78 [mm^2^]. The spacer was devised based on our proposed design guidelines.

Accordingly, after taking account of these conditions, we proposed the new spacer shown in Figure 8, which has the geometric characteristics of *ε* = 0.81, *A_y_/A_mem_* = 0.97 and *A_x_* = 11.78 [mm^2^]. This spacer, hereafter referred to as Spacer 4, consists of a cubic lattice in order to compare the results obtained using the previously tested spacers. It has a staggered arrangement that combines features of Spacers 2 and 3, in which the cubic lattices were shifted by a half lattice in the *z* direction and by a 1/3 lattice in the *y* direction, respectively. Moreover, in order to decrease the contact area between the structure and the membrane, the diameter of the arms extending toward the membrane was halved. These half structures, which are shown as circles in Figure 8, created a t = 0.5 mm space separation that prevents contact with the membrane. It is hoped that the membrane will not be torn by this spacer. 

**Figure 9:**
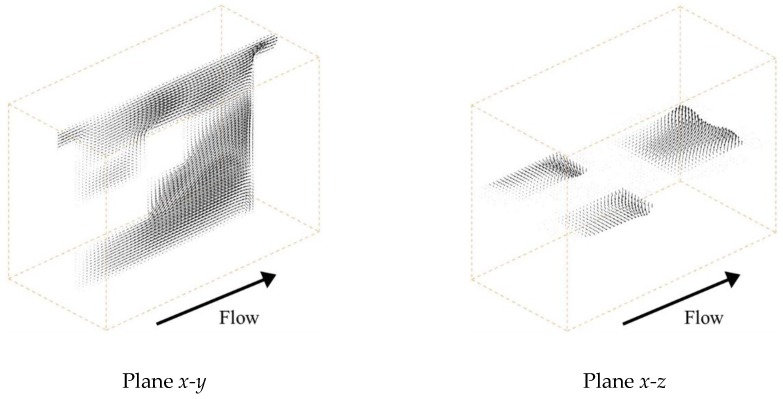
Velocity fields within Spacer 4. Since the structure is shifted vertically in the *y* and *z* directions to the main flow, the flow passing through the spacer is diverted in those directions by the structure.

**Figure 10:**
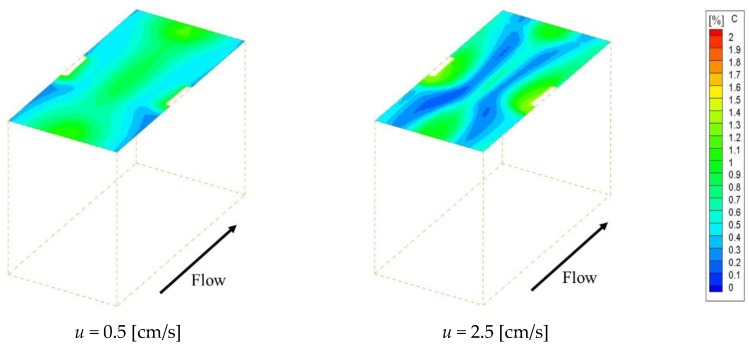
Concentration distribution when using Spacer 4. The spacer 4 can solve the problem that the low concentration region is formed due to the flow stagnation behind the structure.

**Figure 11:**
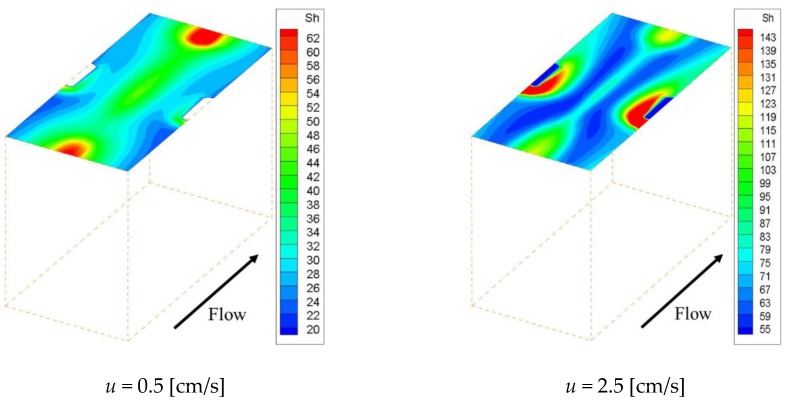
Effective Sherwood number distribution when using Spacer 4. The effective Sherwood number distribution is correlated with the concentration distribution on the membrane. The effective Sherwood number is high at places where the flow is disturbed around the structure, and the overall value is higher than that of the other spacers.

Figure 9 shows the velocity vectors at the centers of the *x*-*y* and *x*-*z* planes, respectively, in the case of a velocity of 1.5 cm/s. Since the structure was shifted vertically in the *y* and *z* directions to the main flow, the flow passing through the spacer was diverted in those directions by the structure. Furthermore, it can be confirmed that the solution is also flowing in the part where the membrane and the structure are separated. This eliminates the flow stagnation on the membrane behind the structure. Figure 10 and Figure 11 show the concentration fields and effective Sherwood number distributions on the ion exchange membrane under velocities of 0.5 and 2.5 cm/s. Interestingly enough, the other spacers had their lowest concentration levels in the flow stagnation behind the structure, but Spacer 4 did not. As can be seen from Figure 11, the effective Sherwood number became high at places where the flow was disturbed around the structure, and the overall value was higher than that of the other spacers.

**Figure 12:**
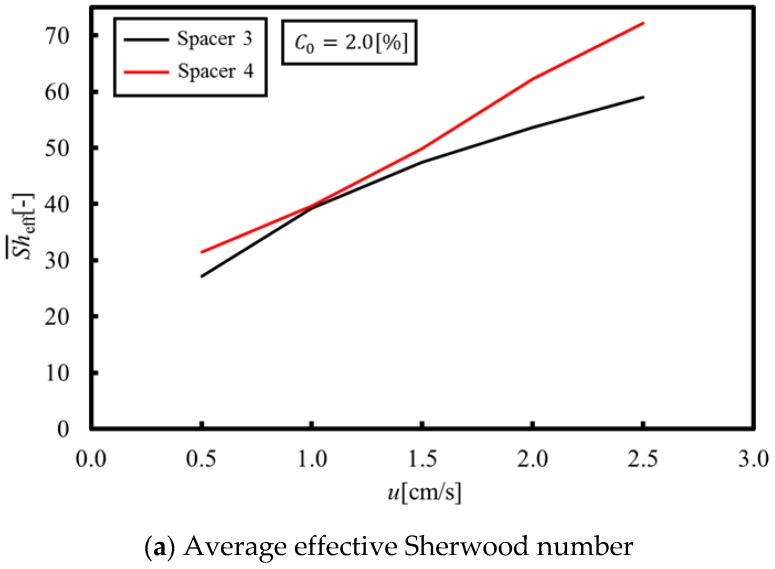

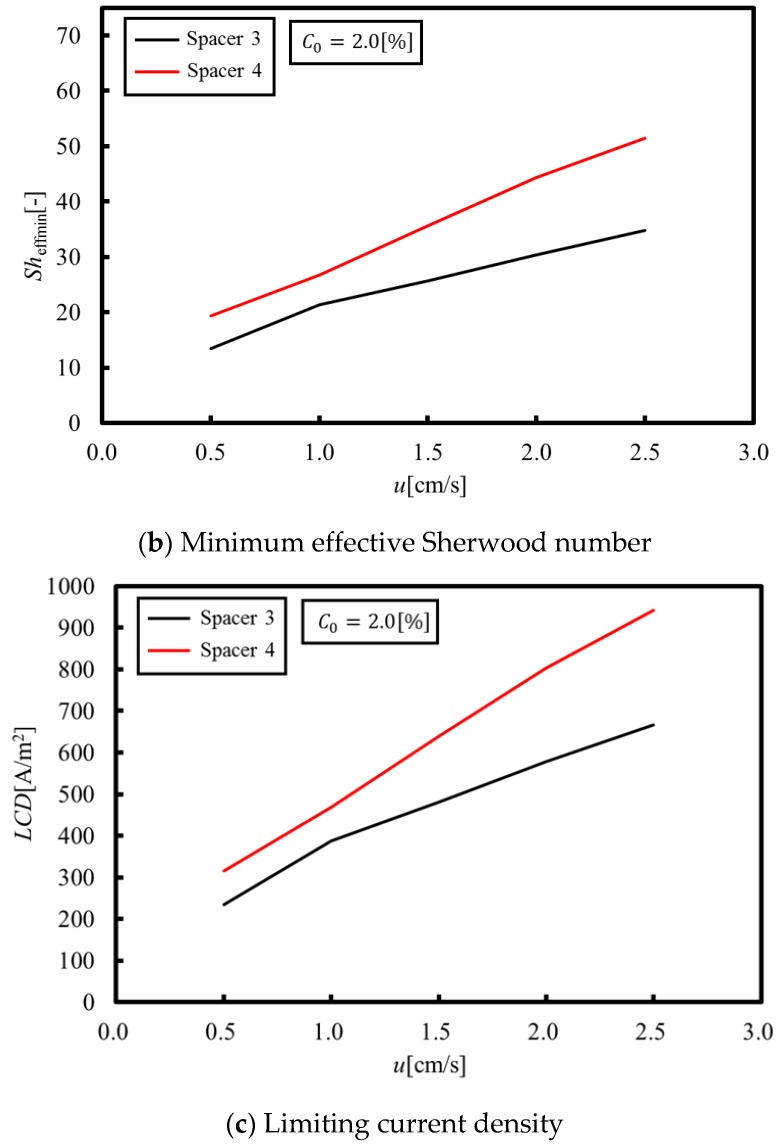
Effect of Spacer 4 on the average and minimum effective Sherwood numbers and on the limiting current density. (**a**,**b**) The average and minimum effective Sherwood numbers are greater than those of Spacer 3; (**c**) The LCD of Spacer 4 is also increased and is higher than that of Spacer 3, as a result of increasing the minimum effective Sherwood number.

Figure 12a,b shows the average and minimum Sherwood numbers under velocities within 0.5–2.5 cm/s, while Figure 12c shows the LCD when Spacer 4 is used. These figures are compared with the result of Spacer 3 showing the highest LCD level. As can be seen from Figure 12a,b, the average and minimum effective Sherwood numbers are greater than those of Spacer 3 because the flow stagnation was eliminated on the membrane behind the structure. Furthermore, as a result of increasing the minimum effective Sherwood number, the LCD of Spacer 4 is also increased and is higher than that of Spacer 3, as shown in Figure 12c. Therefore, we can conclude that the spacer structure design guidelines proposed in this study are effective for increasing the LCD levels. In our future work, we will confirm the effectiveness of Spacer 4 via experiments, and then search for an optimal porous spacer shape that is in accordance with our proposed design guidelines.

## 4. Conclusions

The effects of porous spacers on ion mass transfer were investigated in numerical simulations as a means of increasing limiting current densities. It was found that our proposed numerical method is effective for estimating the minimum value of an LCD, thus providing significant information related to the safe operation of industrial ED systems. Furthermore, we found that the LCD correlates with the minimum effective Sherwood number and that increasing the minimum effective Sherwood number provides a key to increasing LCD levels. Porous spacer design guidelines aimed at increasing the LCD were then proposed based on the results of numerical simulations, after which a new spacer designed in accordance with those guidelines was introduced.

It was found that using a porous spacer structure disturbs the flow on a membrane, which leads to an increase in the effective Sherwood number. Additionally, the LCD levels could be increased by eliminating the flow stagnations that occur behind the structures on the membrane. The LCD of our new spacer (devised based on our proposed design guidelines) was higher than that of the spacers with the highest LCD levels in use at present. Therefore, we can conclude that the proposed design guidelines are effective for increasing LCD levels. In our future research, we will work to further confirm the effectiveness of this new spacer via experiments, and then search for an optimal porous spacer shape that complies with the proposed design guidelines.

## Figures and Tables

**Table 1:** Porous spacer characteristics. (Dimensions of porous spacers, Porosity, Cross sectional intrinsic area at a boundary in the *x* direction *A_x_*, and ratio of cross sectional intrinsic area at a boundary in the *y* direction *A_y_* to total area of membrane *A_mem_*).

Characteristics		Spacer 1	Spacer 2	Spacer 3
Length [mm]	H	5		
	W	3.72		
	L	7.44		
	d	1.28		
Porosity ε [-]		0.62	0.62	0.73
$A_{x}$ [mm^2^]		5.95	5.95	9.08
$A_{y}/A_{mem}$ [-]		0.43	0.43	0.88

## List of Symbols

**Nomenclature**	
*A_mem_*	total area of membrane [m^2^]
*A_x_*	cross sectional intrinsic area at a boundary in the X direction [m^2^]
*A_y_*	cross sectional intrinsic area at a boundary in the Y direction [m^2^]
*c*	molar concentration [mol/m^3^]
*c*_0_	initial concentration [mol/m^3^]
*Δc_B_*	concentration difference between inlet and outlet of a porous spacer unit cell [mol/L]
*d*	arm width of a porous spacer [m]
*D*	diffusion coefficient [m^2^/s]
*F*	Faraday constant (96,485 C/mol)
*h_eff_*	effective mass transfer coefficient [m/s]
*H*	height of a porous spacer unit cell [m]
*i*	current density [A/m^2^]
*L*	length of a porous spacer unit cell unit [m]
*LCD*	limiting current density (LCD) [A/m^2^]
*P*	pressure [Pa]
*q*	molar flax [mol/(m^2^ s)]
*Sh_eff_*	effective Sherwood number [-]
*W*	width of a porous spacer unit cell [m]
*u,v,w*	velocity vectors [m/s]
*u_B_*	intrinsic mean flow velocity [m/s]
*X*	length of dilute channel [m]
*x,y,z*	Cartesian coordinates [m]
*ε*	porosity [-]
*ρ*	density [kg/m^3^]
*ν*	kinetic viscosity[m^2^/s]
**Subscripts**	
*B*	bulk
*W*	wall
*in*	inlet
*out*	outlet
*min*	minimum
1	Na^+^
2	Cl^−^
